# The experience of seeking and accessing help from mental health services among young people of Eastern European backgrounds: A qualitative interview study

**DOI:** 10.1111/papt.12524

**Published:** 2024-03-08

**Authors:** Jerica Radez, Chiara Causier, Daniel Maughan, Felicity Waite, Louise Johns

**Affiliations:** ^1^ Department of Experimental Psychology University of Oxford Oxford UK; ^2^ Oxford Health NHS Foundation Trust Oxford UK; ^3^ University College London London UK; ^4^ Department of Psychiatry University of Oxford Oxford UK; ^5^ Oxford Institute of Clinical Psychology Training and Research, Medical Sciences Division University of Oxford Oxford UK

**Keywords:** barriers and facilitators, early intervention, thematic analysis, youth mental health

## Abstract

**Objectives:**

Most lifetime mental health problems (MHP) start before the age of 25. Yet young people—particularly those of minority backgrounds—often do not seek or access professional help. In the UK, young people of Eastern European (EE) backgrounds represent a large minority group; however, little is known about their experiences of MHP and help‐seeking. In this study, we aim to understand the help‐seeking process from the perspectives of EE young people.

**Design:**

We used a qualitative study design with semi‐structured individual interviews. The results were analysed using reflexive thematic analysis.

**Method:**

Twelve young people (18–25 years) of EE backgrounds, living in Oxfordshire, UK, took part. All participants had experienced a severe MHP and were identified in the community.

**Results:**

EE young people's experiences of MHP and help‐seeking were driven by a sense of being caught between different cultures and simultaneously needing to navigate the potentially contrasting expectations of both cultures. This process was reinforced or tempered by the perceived continuing influence of young people's families, that is, families with more open views about MHP made it easier for young people to navigate through the process of help‐seeking. Young people's internalised cultural and familial beliefs about MHP affected their decision‐making when experiencing difficulties, their levels of trust in services, and perceived sense of resourcefulness and ability to cope.

**Conclusions:**

Recognising and responding to the cultural tension that young people of EE backgrounds may experience can help us to develop more accessible and inclusive mental health services.

## INTRODUCTION

Mental health problems usually start in childhood and adolescence, with the peak age at onset of any mental health problem (MHP) of 14.5 years (Solmi et al., 2021) and nearly three‐quarters of lifetime MHP starting before the age of 25 (Kessler et al., 2007). Prevention, identification, and early treatment of MHP in young people can have a positive impact on their health and well‐being (Marmot et al., 2008). However, young people often do not access appropriate support for their difficulties (Merikangas et al., 2010; Sadler et al., 2018), and access rates are particularly low for young people from non‐White minority ethnic groups (Chui et al., 2021; Coelho et al., 2022: Kataoka et al., 2002). The data also show that young people from non‐White groups access services through different pathways compared with their White counterparts. For instance, young people of non‐White groups are more likely to be referred through social care/youth justice and less likely to access care through voluntary pathways than White young people (e.g. Chui et al., 2021; Edbrooke‐Childs & Patalay, 2019), which is consistent with ethnic disparities in mental health access in adults living in the UK (e.g. Halvorsrud et al., 2018). These findings highlight the importance of understanding and addressing the reasons for these discrepancies. However, less is known about minority White young people and their access to mental health care.

Eastern Europeans (EE) represent a large (>2.2 million) minority in the UK (ONS, 2021). There are many different definitions of Eastern Europe, which is due to a high linguistic, historical and sociocultural heterogeneity of the region. In this study, we used the United Nations Statistics Division (UNSD) definition of Eastern Europe (UNSD, 2021), which is a commonly used scheme of dividing Europe into subregions. Although they represent a large minority in the UK, research suggests that due to the lack of visible differences to their White‐British (WB) peers, EE's experiences of mental health services may be overlooked (Peñuela‐O'Brien et al., 2023). Research studies with adults suggest that cultural differences and mental health stigma associated with historical and socio‐political characteristics of Eastern Europe may continue to shape EE's perceptions of MHP and help‐seeking (Peñuela‐O'Brien et al., 2023; Winkler et al., 2017). Additional barriers/facilitators to help‐seeking in EE communities may include self‐stigmatising beliefs about mental health, (lack) of knowledge of the National Health Service (NHS), (lack of) social support and (lack of) education (Gondek & Kirkbride, 2018). Recent local (e.g. the UK leaving the EU in 2020) and global events (e.g. the Ukrainian war, starting in 2014 and escalating in 2022) may further contribute to higher levels of uncertainty for EEs about their future, as well as xenoracism (i.e. a ‘non‐colour‐coded’ racism, based on someone's immigration status, culture, or religion, Fekete, 2013) targeted at the UK‐living EE immigrants (Rzepnikowska, 2019), and as such, increase their risk of experiencing mental health problems (Schouler‐Ocak et al., 2021). However, to our knowledge, researchers have not yet explored the experiences of MHP in young people of EE backgrounds living in the UK. Understanding the views of EE young people is important, as we know that young people in general experience a wide range of barriers when seeking and accessing professional help, including mental health stigma, a lack of mental health knowledge, negative perceptions of help‐seeking, and a preference for relying on themselves (Radez et al., 2021), and there is some evidence to suggest that the process can be even more challenging for young people of minority backgrounds (e.g. Guo et al., 2015).

In this qualitative interview study, we set out to explore how young people (aged 18–25) of EE backgrounds make decisions about seeking professional help for their MHP and what they see as the main barriers and facilitators in the help‐seeking process. We chose a qualitative approach due to a lack of existing literature in this field. Furthermore, we were particularly interested in experiences and perspectives of EE young people themselves, which can be best captured using qualitative methodology (Hammarberg et al., 2016). The study was set in Oxfordshire, UK, which is a diverse area, and the proportion of people from EE backgrounds in some parts of the county (e.g. Oxford city) is four times higher than in most parts of the country (ONS, 2022). While local community mental health services (e.g. Oxfordshire Early Intervention Service—EIS) report seeing a representative proportion of EE young people with severe MHP (e.g. psychosis) (Oxfordshire EIS, 2023), young people of EE backgrounds seem to be under‐represented at the early stages of MHP (e.g. in Child and Adolescent Mental Health Services—CAMHS) (Oxfordshire CAMHS, 2023), that is, during the peak time for the onset of MHP and key opportunity for prevention and early intervention. Understanding the reasons for this treatment gap could help clinicians to develop more accessible mental health services.

## METHOD

### Design

We conducted a qualitative interview study using Reflexive Thematic Analysis (Braun & Clarke, 2006, 2019) and followed the COREQ checklist (see Supplementary Materials) for explicit and comprehensive reporting of qualitative studies (Tong et al., 2007). The study was granted ethics approval by the University of Oxford Central University Research Ethics Committee (CUREC) (reference R79066/RE001).

### Participants

Participants were recruited in the community. Researchers shared a study flyer with various community stakeholders, including the University of Oxford colleges, Oxford‐based EE societies and associations (e.g. Ukrainian society), and Healthwatch Oxfordshire (i.e. the county's independent health and social care watchdog). Study flyers were distributed in different community venues (e.g. EE supermarkets) across the county and on social media. To help attract eligible participants, the flyer included pictures of relevant EE flags (e.g. Polish, Romanian, Albanian, Ukrainian, Slovakian).

Overall, 14 young people contacted the lead researcher (JR) after seeing the study flyer. One participant subsequently declined to participate, and one participant was not eligible, resulting in 12 participants. Young people were included if they were: (1) aged between 18 and 25 years, (2) identifying themselves as EE (e.g. Polish), (3) having a lived experience of a severe MHP (i.e. mental health issues that significantly interfere with the young person's everyday life, such as severe anxiety, low mood, or psychosis), (4) living in Oxfordshire, (5) sufficient conversational English to engage in the interview and (6) able to provide informed consent. We included participants who were born in the UK or EE.

### Procedure

The lead researcher (JR) first shared a participant information leaflet with potential participants and then assessed their eligibility via telephone. The screening questionnaire consisted of six inclusion criteria, listed above. If eligible, JR then arranged a qualitative one‐to‐one interview at a time convenient for each participant. Each eligible participant was assigned a pseudonym at this stage. The interviews took place remotely (via MS Teams) with an average duration of 50 min (SD = 9 min, range from 42 to 74 min) and participants were reimbursed for their time. All interviews were conducted by JR, a female trainee clinical psychologist with a PhD and formal training in qualitative research methods. Rigorous ethical procedures were followed to ensure that participant benefits were maximised and any risks were minimised. We used an interview topic guide (see Supplementary Materials) flexibly—by adapting the order of questions to the narrative of each participant. Interviews were audio recorded on a password‐encrypted audio recorder. Interviews were transcribed verbatim. Participants were sent a short summary of the study findings after data collection and analysis were completed.

### Public and patient involvement

All study materials (i.e. study flyer, participant information leaflet and interview topic guide) were developed with patient and public involvement (PPI), including young people with a personal experience of MHP and people of EE backgrounds. We also sought PPI input for the study results to ensure that our interpretation of study findings was meaningful for the target population.

### Analysis

Interviews were analysed using six stages of Reflexive Thematic Analysis (Braun & Clarke, 2006, 2019), which was led by JR. In the first stage of analysis, JR familiarised herself with the data, which included transcribing, listening, and re‐listening to the interviews. JR also kept a reflexive journal in which she noted down initial ideas associated with each interview and ideas that emerged during the data analysis (an example of a Reflexive Journal note is available in Supplementary Materials). In the second stage of analysis, JR generated an initial set of codes. This was done by a cyclical and iterative process in which codes were frequently refined and renamed, for example, the codes ‘brought up in a religious environment’ and ‘religious extended family members’ were subsequently refined to ‘affected by religious beliefs’. JR coded for explicit, as well as implicit contents (e.g. a code ‘lack of trust’ was assigned when a participant asked not to be quoted). NVivo (QSR International Pty Ltd, 2021) was used to help organise the codes. The initial set of codes was then reduced by grouping codes in a family of codes (e.g. codes ‘transgenerational trauma in EE’, ‘lack of trust due to the past communist regime’ and ‘austerity in EE countries’ were grouped in a higher order code called ‘EE historical factors affecting perception of mental health’). Similar to the initial set of codes, families of codes were often refined as well. JR, FW and LJ then generated the initial set of themes, which was done by collating families of codes into potential themes. At this stage, we sought connections between the families of codes and attempted different combinations of grouping and regrouping families of codes (see Supplementary Materials for an example of this process) to generate themes. During the process of searching for themes, we paid particular attention to culturally informed themes/subthemes, due to the purpose and focus of the study. Identified themes were then reviewed by JR checking whether the themes were directly related to families of codes, as well as the entire dataset. Connections and relationships between different themes and subthemes were also established by re‐examining participants' narratives about their individual help‐seeking experiences. JR then presented the final set of themes to FW and LJ and they discussed the naming of the themes, as well as their presentation. The names of themes were refined multiple times. In the sixth stage of Reflexive Thematic Analysis, the researchers produced a study report by providing evidence for the generated themes, including supporting quotes.

### Positionality statement

The researchers had extensive previous experience in conducting research on help‐seeking in young people. In addition, the lead researcher shared a similar cultural background to the participants (i.e. Central/Eastern European). The team regularly reflected on the impact of their research interests, previous experiences, and personal experiences on the process of data collection, analysis, and interpretation.

## RESULTS

### Participant characteristics

Table 1 outlines participant characteristics, with participants ordered based on their recruitment order. The Mean participant age was 20.7 years (SD = 1.4 years) and more than half of the participants (58.3%) described themselves as Polish, with the remaining participants being of Slovakian, Romanian and Albanian backgrounds. One participant described themselves as ‘mixed’ background. Table 1 also outlines the participants' main presenting difficulties, with the majority of young people (75%) describing experiences of severe anxiety and/or low mood. Participants varied in terms of their help‐seeking experiences. Notably, most participants who had sought and accessed professional help in their home EE country accessed this via private health care, whereas all participants who reported accessing professional help in the UK managed to do so free of charge (i.e. via the University or NHS mental health services).

**TABLE 1:** Participant characteristics.

Pseudonym	Lived experience of MHP^a^	Help‐seeking experience	
		Type of help	When
Becky	Low mood with suicidal thoughts	Has never sought professional help	
Ben	Low mood	Twice sought and accessed—with a college tutor (UK) and a private psychologist (EE)	Both since starting the university
Leah	Eating disorder (bulimia)	Twice sought and once accessed—an unsuccessful attempt^b^ (UK), a successful attempt (EE)—a private psychologist	Since starting the university (UK), as a teenager (EE)
Natascha	Anxiety, low mood, body dysmorphia	Twice sought and accessed—counselling via the university (UK) and a private therapist (EE)	Since starting the university (UK), as a teenager (EE)
Kate	Anxiety, obsessive‐compulsive disorder, low mood with suicidal thoughts	Sought and accessed multiple times (UK)—specialist mental health services	On multiple occasions since childhood
Dawn	Recurrent depression, social anxiety	Sought and accessed once (EE)—a private therapist^c^	Since starting the university
Wanda	Low mood, history of trauma	Sought and accessed once (EE)—a private therapist^c^	Since starting the university
Amy	Post‐traumatic stress disorder	Sought and accessed once (UK)—GP and counselling via the university	Since starting the university
Shaun	Obsessive‐compulsive disorder	Twice sought and once accessed—an unsuccessful attempt^b^ (UK), a successful attempt (EE)—a private therapist^b^	On multiple occasions since childhood
Bella	Anxiety, low mood	Sought and accessed once (UK)—counselling via the university	Since starting the university
Nick^d^	Low mood	Sought and accessed once (UK)—counselling via the university	Since starting the university
Tina	Anxiety, low mood	Sought and accessed once (UK)—counselling and psychiatrist—via the University	Since starting the university

^a^ Participants' self‐described lived experience of MHP and not a formal diagnosis given by the researchers.

^b^ Participant reported trying to access mental health support, but not being able to successfully receive it due to a variety of reasons (e.g. long waiting times, COVID‐19 pandemic).

^c^ Participant is living in the UK and currently receiving professional help from an EE therapist (remotely).

^d^ Participant asked not to be quoted.

### Themes and subthemes

We identified four themes describing EE young people's experiences of seeking professional help for their MHP: (i) caught between cultures, (ii) continuity of family influence, (iii) informed decision‐making and (iv) sense of resourcefulness. Young people's narratives were led by their sense of feeling caught between their own EE culture and UK culture, and this sense was either further reinforced or tempered by the influences of young people's families. Both internalised cultural and familial beliefs further shaped young people's decision‐making when experiencing MHP and affected their perceived sense of resourcefulness when coping with their difficulties.

**FIGURE 1:**
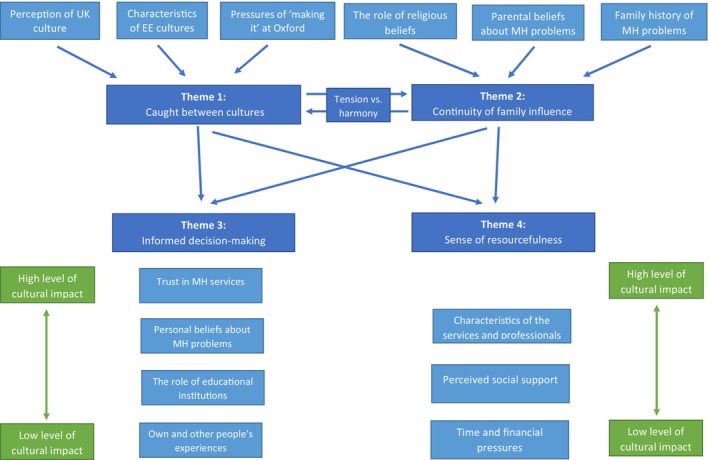
Identified themes and subthemes.

Figure 1 outlines identified themes, subthemes, and the relationship between them, as well as the levels of cultural impact for subthemes identified within themes three and four. Themes with examples of quotes are outlined below. Further quotes are available in Supplementary Materials.

### Theme 1: Caught between cultures

…some people like me can be quite lucky and they will find support in people around them, and people who will tell them ‘no, you're not making this up, it does sound like what you're going through is very difficult, and I feel sorry for you’, but other people may be surrounded by people who are more immersed in the Eastern European culture, which just tells you to toughen up and carry on with your life. So those people might not even realise that what they're experiencing is mental health, and that they should be seeking help for it. (Becky, 20)

This was one of the two central themes describing EE young people's experiences of MHP and help‐seeking. EE young people's perceptions of UK culture, characteristics of EE cultures, which young people carry with them (even upon moving to the UK), and worries around feeling under the pressure of ‘making it’ in Oxford, all contributed to young people's sense of feeling caught between cultures.

Most young people reported perceiving the approach to MH in the UK as more open and normalising compared to their EE home countries, where high levels of stigma and shame around mental health topics were described. Bella (23) said: ‘…here I feel like going to therapy, getting mental health is very normalised now. You know, a lot of like famous people said like they've been going to therapy, and they could help them and it's something normal. While like, yeah, like in Poland I think it will still be like shocking if someone like came, you know, like came out…’. Young people reflected why that might be the case, and they identified certain characteristics of EE communities (e.g. the communities being perceived to be very enclosed, mistrustful) as one of the main reasons. Participants linked these characteristics to relevant historical factors pertinent to EE, such as a lack of financial resources and economic stability, related to the communist regime, as described by Leah (22)—‘…I think my family is quite new to the whole mental health problem thing, because, yeah, they were kind of raised in a, in a kind of sparsity culture, rather than abundance, so there wasn't too much space for these sorts of, I guess they would say next level conversations, that aren't just around physical health and you know, just getting the food and hygiene and that kind of stuff…’. Some young people also reported that their EE native language makes it hard for them to talk about mental health problems. For instance, Natascha (20) found it hard to describe body dysmorphia to her family—‘…I wouldn't even know how to describe this to my family. No, yeah, there's no words that I can use to, you know, openly talk about it, or properly talk about it, or not make it seem like it's nothing, but also not making it seem like it's a very big thing. Yeah, it's a language barrier…’. Some participants reported finding it difficult to express their MH concerns to their families upon their moving to Oxford—due to feeling ‘guilty’ for not fulfilling family expectations and fully embracing the opportunities they have by studying in such an ‘elite’ place. Dawn (21) said: ‘I think quite a few of us get imposter syndrome when reaching out for help, especially since, yeah, in Oxford like…we have such a privileged background now and maybe there's like survivors’ skills often of like, you made it out the country, you did all these things, you look back home, and like the rest of my family's not doing that well economically and like, it's like ‘well why I am complaining?’, so…’. This illustrates how young people's pressures of needing to fulfil their own, parental, and cultural expectations can act as a barrier in seeking professional help for their MHP.

### Theme 2: Continuity of family influence

…I had friends with mental health issues, and I remember being 15 and finding out that one of my closest friends was cutting, being very confused about what that meant, talking to my parents about it and my parent's response was, ‘you shouldn't be friends with her anymore’. So, I think that kind of, again, influenced my views about my own mental health. (Wanda, 20)

Continuity of family influence was the second central theme in EE young people's narratives, and it describes how participants' experiences of mental health problems are shaped by their primary families. Notably, these influences can either exacerbate or temper young people's sense of feeling caught between different cultures.

Participants differed in terms of what beliefs their parents held around mental health, with some participants reporting their parents seeing MHP as a sign of weakness, whereas other participants, like Shaun (23) seeing their parents as very open to these topics—‘…Yeah, so, so my family, well the younger generation is, well understand it [mental health] pretty well. Well, especially my parents and they have been very supportive throughout my whole life really’. Some participants also reported a history of MHP within their family, and notably, this usually represented a barrier in EE young people's help‐seeking—in particular if parental experiences were associated with shame, stigma and fears of potential repercussions, as described by Dawn (21) ‘I wanted to go to some kind of psychologist, but my dad was very much against it… He told me that they would write up everything I say and then I won't be able to get a job because that's how it looked in his times’. Finally, this theme captures the interplay between religious beliefs within the primary family, extended family, and wider social context, and participants' experiences of mental health issues. Most participants reflected on the (mainly negative) role of traditional religious beliefs on help‐seeking in their home country. For instance, Amy (19) described the sense of guilt in relation to young people's experiences of depression due to someone's own responsibility for becoming unwell being imposed by the Catholic church—‘I've like heard, less so my aunties and uncles, but like my Grandparents, like if someone's suffering they're like, oh ‘they were probably a bad person, they've been sinning a lot and this is God's punishment for them’.

### Theme 3: Informed decision‐making

… I grew up in the same school since I was 6 until I graduated at 18, so, erm, I knew a lot of the teachers, and although they must have noticed that something was kind of wrong or something, no one really called it out, accept for one like PE teacher who was also like my friend, she said it once, and I don't think it had an impact on me too much… (Leah, 22)

EE young people's decision‐making when experiencing mental health problems was informed by the continuing influence of their family, as well as their sense of feeling caught between cultures, leading to some barriers and facilitators identified within this theme being more (e.g. trust in MH services) and some less (e.g. own and other people's experiences) culturally informed than others.

Some EE young people reported feeling mistrustful about (mental) health services in their own country, which could act as a barrier when considering help‐seeking in the UK. Tina (20) said: ‘I wouldn't have seen anyone in Romania, like any therapist…there's notorious problems with [the] Romanian health system, you know, like sort of, if you get lucky you will be treated very well and, you know, there will be no problem, but if you're unlucky, you know, then you might be worse off than at the start’. Young people also talked about the role of various stakeholders (e.g. primary/secondary schools, universities, parents, friends, and partners) in their understanding of their mental health difficulties and making decisions to seek help, such as recognising symptoms of MHP and knowing where to get help. Related to that, the theme also captures how young people's personal appraisals of MHP can act as a barrier or facilitator to help‐seeking, as described by Ben (20), who decided not to seek help: ‘I thought, and still kind of do, but not to that extent, that, I still kind of believe, that like mental problems, that I have at least, were not like purely medical things, but they were some things I was able to figure out myself.’. In addition, young people's own or other people's (lack of) experiences of mental health and help‐seeking can further inform their help‐seeking decision‐making process. For instance, Kate described being affected by a negative experience of professional help: ‘…I think, objectively, some of the stuff that was said, or the kind of attitudes that were shown were a bit questionable. One time, I was in hospital for a suicide attempt and one of the Crisis Team said to my mother, ‘oh, she'll be back again soon’ and that didn't really feel like a great message to be receiving when I was already at a very low point’.

### Theme 4: Sense of resourcefulness

…If it was like a Polish therapist that had like typically Polish cultural religious mindset, I don't think I'd feel comfortable talking to them. But if it was someone who was aware of like the typical Polish cultural mindset and they opposed it, so they like experienced it, but also understood the importance of seeking out mental health support, I'd probably feel more comfortable speaking to them than someone who, like, had no idea like an English therapist… (Amy, 19)

Two central themes—caught between cultures and continuity of family influence—also shaped EE young people's sense of resourcefulness when experiencing mental health problems and seeking/accessing help.

An important culturally informed factor affecting young people's sense of resourcefulness is perceived characteristics of mental health services, such as (lack of) their cultural awareness. Kate (19) said: ‘…I think a lot of British professionals don't have a great awareness that it's not as simple as ‘go talk to your parent’. It's not as simple as like, ‘have this conversation, start it…’, you know, that's not a feasible answer for a lot of people because sometimes the cultural differences can be massive and if you've never, ever had a conversation, it's really difficult’. Some participants also reported professionals' characteristics that might facilitate their help‐seeking, such as a preference for therapists of their gender and age. Young people's perceived social support (e.g. support by their partners, friends) was also identified as a significant contributor to the sense of resourcefulness, and notably, young people valued this support even if their close ones were living abroad (i.e. in their home EE countries). Tina (20) described an experience of her social support facilitating help‐seeking: ‘…I think a friend told me, like…he told me, sort of, ‘maybe you should like talk to someone’, you know, ‘because this is worrying’. It's just like, you know, ‘it seems like quite bad’. And I think that I agreed with him’. Finally, young people named time and financial pressures as important factors affecting their sense of resourcefulness, with the majority of young people identifying their own and professionals' lack of time and availability, as well as high costs of private support in the UK as one of the barriers to accessing help. Amy (19) said—‘…there's some who are able to afford it, they can like seek out help privately if like, private therapy and stuff but that's not accessible for everyone. I know that when I first like sought help I did get a lot of resources from the welfare team at college and quite a few of them were like paid, that I'd have to pay for therapy, or like some sort of counselling workshops and stuff, that's something that's definitely not accessible to me’. Notably, all participants who reported receiving private therapy did this remotely with a therapist from their own country.

## DISCUSSION

In this study, we set out to understand how young people of EE backgrounds living in the UK make decisions about help‐seeking for their MHP, and what they perceive as the most common barriers and facilitators in this process. To our knowledge, this was the first study investigating the views of young EE people on this topic in the UK. We identified a number of culturally informed barriers to seeking professional help, such as: high stigma and shame associated with mental health problems, lack of trust in (mental) health services, the tendency of EE communities to keep things private (i.e. within the family), strong religious beliefs that can reinforce someone's sense of guilt for experiencing MHP, and transgenerational ‘negative attitudes’ towards mental health and help‐seeking (e.g. self‐reliance and not seeing MHP as being as serious as physical health problems) in EE cultures.

The results of this study also highlight young people's need to navigate the (often conflicting) relationship between the expectations of their EE culture and British culture. However, a few young people reported very open views about mental health in their immediate families, and some of these young people contributed this openness to their parents belonging to a younger generation and hence being less affected by traditional views of mental health. Generational differences in people's understanding of MHP were often discussed by young people, and nearly all of them described older generations finding these topics particularly stigmatising. Participants said that one of the reasons for large generational differences might be related to older generations growing up in times of atrocities related to the Cold War, which made people particularly mistrustful and unlikely to talk about contentious topics like mental health. For instance, one of the members of the PPI said that the mental health services in their own country would historically use the information about the service users' MHP to ‘lock them up’ and ‘take their babies away from them’. The same person also reflected on how the fears of negative consequences of disclosure projected to other figures of authority (e.g. schools and parents), which could make young people from EE communities less likely to disclose their problems to their parents/teachers. These findings are also in line with previous research, which suggests that the lack of trust in mental health services in Eastern European communities can be attributed to historical political factors (e.g. communism), where disclosing mental health issues to the authorities could have detrimental repercussions, including deportation (e.g. Doblytė, 2022). Notably, all these societal and familial cultural differences affected young people's decision‐making when struggling with their own MHP. In particular, the experience of lack of trust in (mental) health services in their EE countries might sometimes act as a barrier to trusting the services in the UK. Finally, young people's cultural views and expectations also informed their perceived sense of resourcefulness when seeking help, with some young people reflecting on a concern that British therapists may not necessarily understand EE young people's background, which may have affected their decisions to not seek help.

In addition to many culturally specific barriers to help‐seeking, outlined above, EE young people in our study also identified a range of other, more general barriers, such as logistical barriers (e.g. lack of time and money) and preference to rely on themselves when facing mental health difficulties. This is consistent with the key findings of systematic reviews on young people's help‐seeking for a wide range of mental health problems (Gulliver et al., 2010; Radez et al., 2021). However, to our knowledge, these systematic reviews did not include any studies focusing on the experiences of EE young people, which highlights the importance of the qualitative approach in unpicking the cultural nuances pertinent to this group of young people. Finally, there was a trend in young people seeking and accessing private support in their home EE countries (e.g. Poland) rather than in the UK. This is consistent with previous research including Polish adults living in the UK, who commonly reported a preference for seeking and accessing health services in Poland rather than in the UK (Osipovič, 2013). However, to our knowledge, no previous research has yet reported this preference in young people EE experiencing MHP.

Our study has clear clinical implications. Based on the views of the young people interviewed, clinicians might find it beneficial to adopt a curious approach when working with young people of EE backgrounds. For instance, clinicians might find it helpful to ask young people about their family background and how it may have affected their relationship with MHP and help‐seeking. This is consistent with previous research which suggests that discussions about people's backgrounds can lead to an increase in mental health treatment participation in minority ethnic patients (Aggarwal et al., 2016). In terms of fostering trust in mental health services, young people suggested that clinicians could ensure they clearly explain what to expect from mental health support and how their information will be used. Clinicians might also find it helpful to promote a culture of safety, dignity, compassion and avoidance of stigma and coercion to help build trust in services in EE communities (Gaebel et al., 2014). Fostering trust is particularly important as it may lead to higher levels of satisfaction with the services and consequently, increase the likelihood of service usage in the future (Chang et al., 2013). Young people might also appreciate clinicians' awareness of socio‐political events pertinent to EE countries (e.g. wars, communism) that can affect the relationship of young people, and their support network, to mental health, as well as contribute to some specific mental health issues within the family (e.g. transgenerational trauma). Finally, practitioners might incorporate this knowledge in their direct clinical work with EE young people by using culturally adapted interventions, which have been shown to provide benefits to intervention outcomes (Sue et al., 2009).

In terms of wider systemic and service implications, participants in our study suggested that offering a choice of therapists (i.e. therapists of the same or different backgrounds) might help them navigate through the cultural tensions and make informed decision‐making about help‐seeking. This lack of a fixed rule to match minority therapists with minority service users has been also reported in previous research (Gurpinar‐Morgan et al., 2014). In addition, services should aim to offer therapy in different languages, which might be particularly important for young people who are less well educated. All participants also reported appreciating the opportunity to take part in the research study focusing on EE communities, and therefore, one of the strategies for increasing the levels of trust and engagement with mental health services in EE communities might be including EE young people in the commissioning, planning, and delivery of mental health services (co‐production). Furthermore, given the under‐representation of young people in CAMHS, as suggested by the local audit of referrals, it may be particularly beneficial to co‐produce the services with young people and their families. Mental health services, as well as educational institutions, such as schools and universities, can also aim to improve young people's knowledge of MHP and reduce mental health stigma in EE communities through outreach activities (e.g. mental health talks in community centres, places of worship) and by involving members of EE communities in research.

### Limitations

This study has several limitations. Firstly, all participants were university students, and most of them self‐identified as being from privileged backgrounds, meaning that the participants in this study will not be representative of the population of EE young people in the region. The study sample size was also relatively small, and more than a half of the sample identified as Polish. The invitation to participate was circulated in a number of community venues and channels, and it is possible that the lack of response from non‐student EE young people reflects higher levels of mental health stigma in this population and other (e.g. language) barriers. Another limitation of the study relates to language barriers. As the study flyer was only available in English, and as there was no option to conduct the interview with an interpreter, the study may excluded EE young people who do not speak English and maybe those less comfortable with speaking in English. Furthermore, no participants had an experience of psychosis, which might indicate a particularly strong stigma around more serious MHP in EE young people. Finally, it is important to acknowledge the researchers' previous experience and interests in understanding and facilitating help‐seeking in young people, which might have led them to interpret the results of the current study through the lenses of previous research.

## CONCLUSIONS

Help‐seeking in young people is a complex process and being a young person of a minority background can further complicate this process. Young people of EE background represent a significant minority group in the UK, and due to a lack of visible difference to their WB peers, their experiences of seeking and accessing professional help for mental health problems may be overlooked. The results of this study suggest that EE young people can experience a range of culturally influenced barriers in the process of seeking and accessing mental health support. One of the most common barriers was related to young people's need to navigate the views of their own, as well as UK culture, and the cultural views of their own culture being further imposed by their immediate families. Mental health professionals have a responsibility to maintain a curious and open approach to young people of EE backgrounds and explore their culturally informed views and expectations in relation to mental health and help‐seeking. It is particularly important for the practitioners, as well as the services, to work on fostering trust in mental health services in young people of EE. In addition to mental health services, young people in this study also suggested that educational and research institutions could reduce the barriers to help‐seeking by improving young people's knowledge about common MHP and available help and reducing mental health stigma. Finally, young people in this study were clear about the importance of their views being considered when talking about making mental health services more accessible. Including young people through co‐production might be the key to developing more inclusive and easily accessible mental health services in the future.

## AUTHOR CONTRIBUTIONS

**Jerica Radez**: Conceptualization; methodology; formal analysis; investigation; writing – original draft; writing – review and editing. **Chiara Causier**: Conceptualization; methodology; writing – original draft. **Daniel Maughan**: Conceptualization; methodology; writing – original draft. **Felicity Waite**: Conceptualization; methodology; writing – original draft; writing – review and editing; supervision. **Louise Johns**: Conceptualization; methodology; writing – original draft; writing – review and editing; supervision.

## CONFLICT OF INTEREST STATEMENT

The authors have declared that they have no competing or potential conflicts of interest.

## ETHICS STATEMENT

The study was granted a full ethics approval by the Oxford University Research Ethics Committee (CUREC) (reference R80946/RE001). All participants were required to provide an informed consent prior to taking part in the study.

## Supporting information


Data S1.

## Data Availability

The full dataset that supports the findings of this study (i.e. participants’ interview transcripts) are not publicly available due to privacy or ethical restrictions. Parts of the dataset (e.g. anonymous codes) are available from the corresponding author upon reasonable request.
