# Supplementary material for: The experience of seeking and accessing help from mental health services among young people of Eastern European backgrounds: A qualitative interview study
Source: Psychol Psychother. 2024 Mar 8;97(3):425–38. doi: 10.1111/papt.12524 (PMC11571270; doi:10.1111/papt.12524)
Supplement: Supplementary file 1 — Data S1. [file PAPT-97-425-s001.docx]

**The experience of seeking and accessing help from mental health services amongst young people of Eastern European backgrounds: A qualitative interview study.**

*Help-seeking in Eastern European young people*

Supplementary Materials

**S1 – COREQ (Consolidated criteria for reporting qualitative studies) Checklist**

| **No. Item** | **Guide questions/description** | **Reported on Page #** |
| --- | --- | --- |
| **Domain 1: Research team and reﬂexivity** |  |  |
| *Personal Characteristics* |  |  |
| 1. Inter viewer/facilitator | Which author/s conducted the inter view or focus group? | 6 |
| 2. Credentials | What were the researcher’s credentials? E.g. PhD, MD | 6 |
| 3. Occupation | What was their occupation at the time of the study? | 6 |
| 4. Gender | Was the researcher male or female? | 6 |
| 5. Experience and training | What experience or training did the researcher have? | 6-8 |
| *Relationship with participants* |  |  |
| 6. Relationship established | Was a relationship established prior to study commencement? | n/a |
| 7. Participant knowledge of the interviewer | What did the participants know about the researcher? e.g. personal goals, reasons for doing the research | 6 |
| 8. Interviewer characteristics | What characteristics were reported about the inter viewer/facilitator? e.g. Bias, assumptions, reasons and interests in the research topic | 8 |

| **Domain 2: study design** |  |  |
| --- | --- | --- |
| *Theoretical framework* |  |  |
| 9. Methodological orientation and Theory | What methodological orientation was stated to underpin the study? e.g. grounded theory, discourse analysis, ethnography, phenomenology, content analysis | 8 |
| *Participant selection* |  |  |
| 10. Sampling | How were participants selected? e.g. purposive, convenience, consecutive, snowball | 6 |
| 11. Method of approach | How were participants approached? e.g. face-to-face, telephone, mail, email | 6 |
| 12. Sample size | How many participants were in the study? | 9 |
| 13. Non-participation | How many people refused to participate or dropped out? Reasons? | 6 |
| *Setting* |  |  |
| 14. Setting of data collection | Where was the data collected? e.g. home, clinic, workplace | 6 |
| 15. Presence of non-participants | Was anyone else present besides the participants and researchers? | n/a |
| 16. Description of sample | What are the important characteristics of the sample? e.g. demographic data, date | 9-10 |
| *Data collection* |  |  |
| 17. Interview guide | Were questions, prompts, guides provided by the authors? Was it pilot tested? | 6-7 |
| 18. Repeat interviews | Were repeat inter views carried out? If yes, how many? | n/a |
| 19. Audio/visual recording | Did the research use audio or visual recording to collect the data? | 7 |
| 20. Field notes | Were ﬁeld notes made during and/or after the inter view or focus group? | 7 |
| 21. Duration | What was the duration of the inter views or focus group? | 6 |
| 22. Data saturation | Was data saturation discussed? | n/a |
| 23. Transcripts returned | Were transcripts returned to participants for comment and/or correction? | n/a |
| **Domain 3: analysis and ﬁndings** |  |  |
| *Data analysis* |  |  |
| 24. Number of data coders | How many data coders coded the data? | 7 |
| 25. Description of the coding tree | Did authors provide a description of the coding tree? | 7 |
| 26. Derivation of themes | Were themes identiﬁed in advance or derived from the data? | 7-8 |
| 27. Software | What software, if applicable, was used to manage the data? | 7 |
| 28. Participant checking | Did participants provide feedback on the ﬁndings? | 7 |
| *Reporting* |  |  |
| 29. Quotations presented | Were participant quotations presented to illustrate the themes/ﬁndings? Was each quotation identiﬁed? e.g. participant number | 11-16, SM |
| 30. Data and ﬁndings consistent | Was there consistency between the data presented and the ﬁndings? | 11-16 |
| 31. Clarity of major themes | Were major themes clearly presented in the ﬁndings? | 11-20 |
| 32. Clarity of minor themes | Is there a description of diverse cases or discussion of minor themes? | 11-20 |

**S1 – Interview Topic Guide**

**Introduction:** *Many young people experience mental health problems. However, they can often find it hard to get help. In this interview, I will be interested to hear more about you, your experiences, and ideas for better helping young people when they experience mental health problems.*

*This interview will last approximately one hour and there will be no right or wrong answers. I will only be interested in what you think.*

**Understanding of SMHP**

1. Can you tell me about your understanding of mental health problems?

- Probe: what does ‘having a serious mental health problem’ mean to you?
- Probe: how can you tell if someone of your age is struggling with serious mental health problems?

1. How do people in your community view mental health problems?

- Probe: how do people in your family see serious mental health problems?
- Probe: how do your friends perceive serious mental health problems?
- Probe: what makes you/your family/friends think about serious mental health problems in a certain way?

**Personal experience of SMHP and help-seeking**

1. Can you tell me about your experience with mental health problems?

- Probe: who played the most important role in helping you identify symptoms of a serious mental health problem in you?
- Probe: what role did your family play in identifying symptoms of a serious mental health problem in you?
- Probe: who are your friends? what role did your friends play in identifying symptoms of a serious mental health problems in you?
- Probe: what role did school/college/work play in identifying symptoms of a serious mental health problems in you?
- Probe: do you have any religious beliefs? If so, what role did these play in identifying symptoms of a serious mental health problem in you?

1. Have you sought *any* help for your symptoms of serious mental health problems?
2. *If yes – can you describe this experience to me*?

- Probe: where did you seek help? Who did you speak to?
- Probe: what made you speak to that particular person?
- Probe: have you sought help with professionals (including schoolteacher or your GP)?

1. *If no – What stopped you from seeking help?*

- Probe: what stopped you from speaking with your family/friends?
- Probe: what stopped you from speaking to a professional (including school teacher or your GP)?
- Probe: what role did you family/friends play in you not speaking to a professional?

**Attitudes towards help-seeking**

1. What do you think about speaking to other people about symptoms of serious mental health problems?

- Probe: Some people think it is brave to speak about symptoms of serious mental health problems, others think the opposite. What do you think?
- Probe: Who do you think plays the most important role in how you see help-seeking for serious mental health problems? School/friends/family? Why?
- Probe: What would you think about someone else who speaks about their mental health problems to other people?

1. Do you think that you would think about help-seeking differently if you were living in another country (e.g., Poland – *adapt based on participant’s nationality*). *If yes* – how do you think that living in a different country affects your thoughts about help-seeking?

**Barriers to help-seeking**

1. Now let’s think about professional help only. What do you think are the main reasons that stop young people like yourself from seeking professional help for symptoms of serious mental health problems?

- Probe: How easy/hard it is for young person like yourself to get professional help for symptoms of serious mental health problems?
- Probe: Would your friends know where and how to seek and access professional help for symptoms of serious mental health problems?
- Probe: What role does a family/friends play in young person seeking and accessing professional help for symptoms of serious mental health problems?
- Probe: What role does someone’s cultural background/nationality play in young person seeking and accessing professional help for symptoms of serious mental health problems?

**Facilitators to help seeking**

1. In Oxfordshire, many people like yourself do not seek professional help for their serious mental health problems. What do you think are things that could make it easier for young people like yourself to seek or access professional help?

- Probe: How would you want the professional services to look like?
- Probe: What kind of services do you think would be most interesting/attractive for young people like yourself?
- Probe: what kind of person would young people like yourself find it easiest to talk to? What role does the professional’s nationality play in that?
- Probe: How do you think that people around you could help you seek/access professional help?
- Probe: what do you think that professionals can do to make the services more available and accessible for young people like yourself?

**Ending**

1. Finally, is there anything we haven’t discussed and you think is relevant for the purpose of this discussion?

**S3 – Examples of notes from Reflective Diary**

| **Event** | **Personal reflection** |
| --- | --- |
| Participant describing only realising that a school psychologist existed in their school years upon finishing. | Remembering that there were two school psychologists in my school, but they were only dealing with ‘naughty children’. Going to a psychologist to speak about anxiety and depression was never something anyone considered doing. |
| Participants describing EE beliefs about ‘fixing’ things, including mental health problems. | Thinking about the experiences in my own country, especially when talking to older generations, they are often reluctant to speak about mental health issues, and offer practical solutions instead (e.g., going for a walk). |
| Participants describing feeling privileged to come from a background that is supportive and remains supportive even upon them moving to the UK. | Reflected on my own personal experiences of always feeling supported by my primary family, even upon moving to the UK, and thinking about the advantages of being in this position. |
| Organising codes – i.e., grouping initial set of codes in the families of codes | Had ideas/names for families of codes in my mind from conducting another study on help-seeking. Had to actively push that prior knowledge to the side and think more about the current project and its novelties. |

**S4 – Example of grouping and re-grouping of family of codes**

**
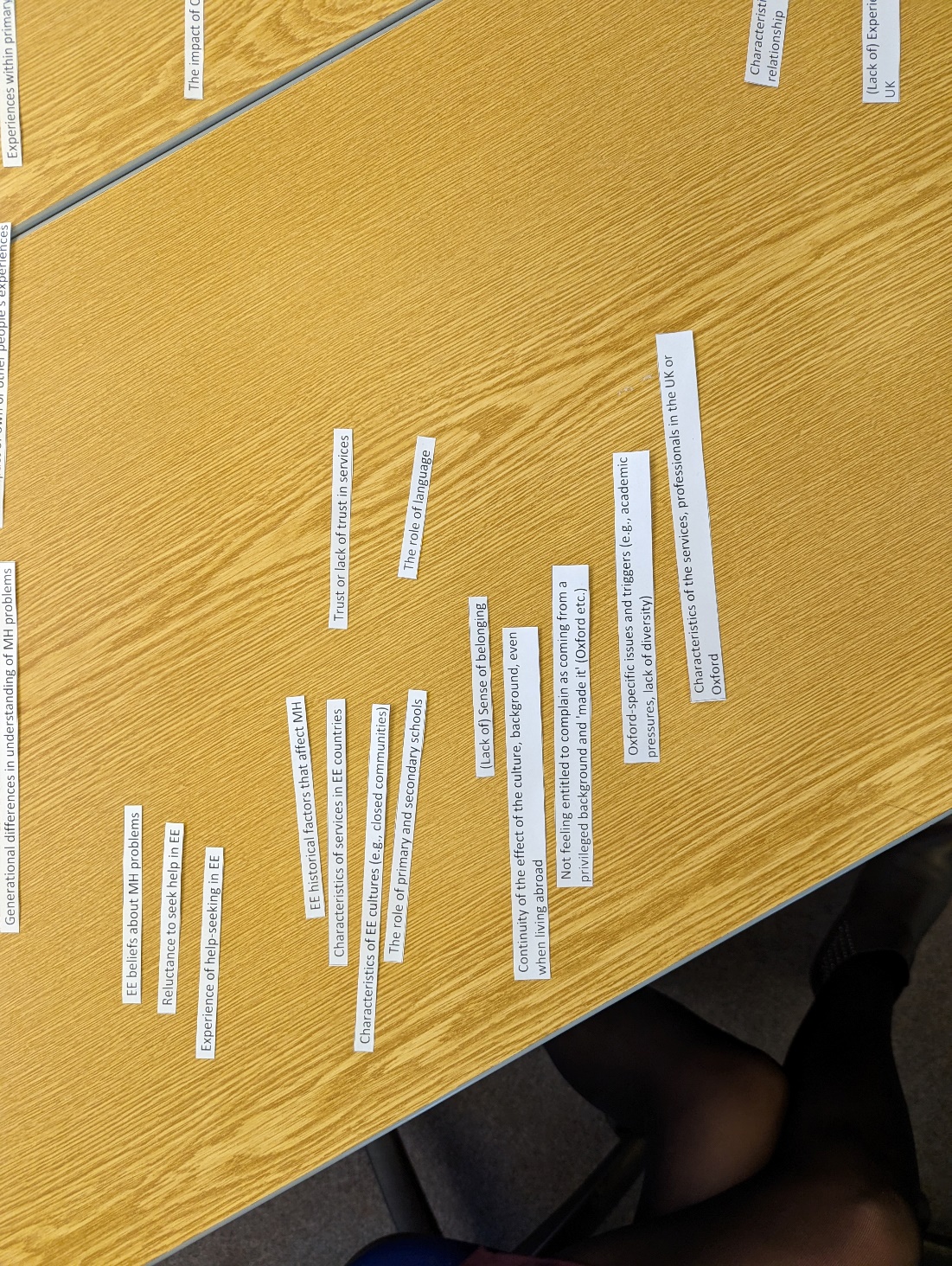

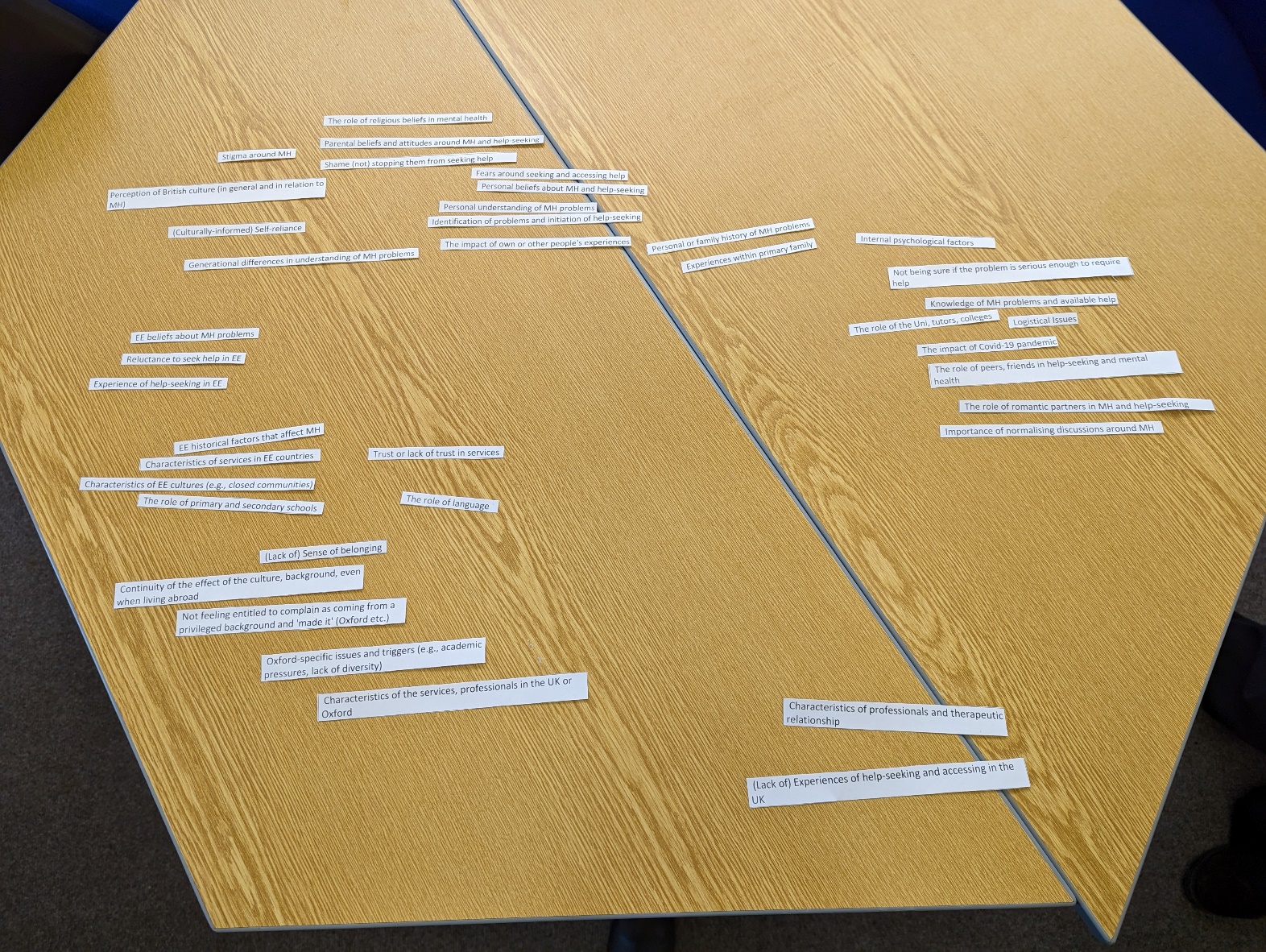
**

**S5 – Additional Quotes**

| **Theme 1: Caught between cultures** | |
| --- | --- |
| Perceptions of British culture | *But here, you say you're anxious, there are something that, I mean, people listen and not only that, people kind of try to give you solutions in a way, or like try to be like 'hey, you could talk to someone' or 'hey, you can do this' or 'hey you can do that' , but they don't necessarily try to ask or justify it, which I think is very mindful.* (Natascha) |
| Characteristics of EE cultures | *I think it's largely sort of the heritage of it [mental health] not having been a topic when they were growing up, and I think that in, in let's say the East, we didn't  go through this, let's call it  'revolution' that the West did, that it became an issue that was talked, that it became an issue a focus for the NHS, for example, we don't have that so we just carry  on with this, you know, sort of archaic approach of not really talking about mental because everyone is sad from time to time, erm, yeah so I really think it's a cultural thing.* (Becky) |
| Pressures of 'making it' at Oxford | *Another factor is you...and that’s something even I have felt with my pretty open attitude that I can be letting my family down cause, you know, they’ve cared for me that well and they’ve given me everything that I possibly could have wanted, and they were very supportive and then I still have a lot of stupid mental health problem, and I felt that well, I’m not, you know, I’m not being grateful enough to my parents because I’m, well, clearly I could be, you know, depressed or have OCD if I was raised in a house where they didn’t support me, but I was raised in a fantastic house.* (Shaun) |
| **Theme 2: Continuity of family influence** | |
| Family history of MH problems | *I could never suggest like to my Grandma, for example, 'oh you should go to therapy' because she would be offended. And I don’t know like my Mum, which is kind or weird like, she had told me like recently she thinks my Dad has like a bipolar disorder, which was like, she should not diagnose that. But like no-one ever diagnosed that, but I know that no-one ever will, because my Dad will not go to like a psychologist or psychiatrist. Because like it is perceived, you know, as if there's something like, wrong with you.* (Bella) |
| Parental belief about MH problems | *I think it was more like, 'you can live with it, it’s not bad enough to stop you doing stuff', because like…and I guess he [father] did seek help and he said it didn’t hep him and he’s still doing all this stuff, and I guess, I get it, like he did give up his job, you know, he left his friends and that to come to this country and that’s gonna create a certain attitude in you.'* (Kate) |
| The role of religious beliefs | *I know the access to mental health services in Poland is really bad and definitely not taken seriously, plus also the very like conservative religious minds that would definitely have more blame put on to me for being raped. It wouldn’t be considered, like, everyone I’ve spoken to [in the UK] are like, 'oh it’s not your fault'. But I don’t think this is what I’d be met with in Poland.* (Amy) |
| **Theme 3: Informed decision-making** | |
| Trust in MH services | *I think it’s more the culture itself and for me it was repercussions of seeking help. Unfortunately sometimes there are repercussions of, I dunno, if there was this huge view that was instilled of me of nobody’s gonna want to hire me and this is gonna be like a huge issue for the rest of my life. And if you go in already struggling and thinking that you are-- it does-- it feels like it’s such lose, lose situation because you reach out and you already don’t trust services because you’ve been taught to not trust the services. There is this view of like crazy people and you get locked up...* (Wanda) |
| Personal beliefs about MH problems | *Well I think that the biggest one is like just being kind with yourself and allowing yourself to accept that you feel that way cause I feel like I do it also sometimes. Like when I feel bad or like, you know, I have bad thoughts, it was like difficult for me, I always tell myself like it’s my fault, I’m just lazy, I just don’t want to do my work and I think that it’s accepting that you can get help.* (Bella) |
| Own and other people's experiences | *So, yeah, basically that was the point where I really tried to get help. It was so bad I actually ended up asking a priest for help because, you know, everywhere was booked out, I mean, I went to college counsellor, but you know how they are completely booked out... So, then I’m talking to a pastor about it which is just terrible.* (Tina) |
| The role of educational institutions | *Maybe like at the beginning when they [university students] just join, to have some kind of introduction like, you know, we have a lot of introduction course like talks in the first week. Maybe have like 10, 15 minutes talk about mental health kind of... I forgot the nice word, but kind of like make it more accessible, like explaining what it is. Kind of trying to like get rid of all this bias that people from this country [Poland] may have.* (Bella) |
| **Theme 4: Sense of resourcefulness** | |
| Characteristics of the services and professionals | *...maybe things like, un-understanding of people's backgrounds, erm, because, especially in the UK, the diversity of the professionals makes quite a big difference, because you have people coming from all sorts, from all sorts of countries, backgrounds, like economic statuses, I'm sure that makes a difference. Erm, and like therapist match.* (Leah) |
| Perceived social support | *Yeah, well I have a good friend who’s here in Oxford as well and erm he’s always... I’d say, very smart guy, and he has a very similar perspective to me, erm when it comes to life let’s say. So when we talk to each other about this topic, sometimes, like we will understand each other really well, like I feel I would be better if I talk to this guy...So we are both confident that a different perspective [professional] would be helpful, but…* (Ben) |
| Time and financial pressures | ...*I always wanted – in Oxford – to be a place that...you just go there, you just have some tea and somebody to talk to because otherwise you’re all the time alone, in your room or in the study session... and it would be just nice to have some place where you could talk to somebody face to face or to someone that is going through the same stuff you do... So, being able to just sit somewhere, go somewhere… yeah*. (Dawn) |
